# Cartography of Methicillin-Resistant *S. aureus* Transcripts: Detection, Orientation and Temporal Expression during Growth Phase and Stress Conditions

**DOI:** 10.1371/journal.pone.0010725

**Published:** 2010-05-20

**Authors:** Marie Beaume, David Hernandez, Laurent Farinelli, Cécile Deluen, Patrick Linder, Christine Gaspin, Pascale Romby, Jacques Schrenzel, Patrice Francois

**Affiliations:** 1 Genomic Research Laboratory, Infectious Diseases Service, Geneva University Hospitals, Geneva, Switzerland; 2 Fasteris SA, Plan-les-Ouates, Switzerland; 3 Department of Microbiology and Molecular Medicine, University Medical Center, Genève, Switzerland; 4 INRA, UBIA and Plateforme GenoToul Bioinfo, UR 875, Castanet-Tolosan, France; 5 Architecture et Réactivité de l'ARN, Université de Strasbourg, CNRS, IBMC, Strasbourg, France; National Institute of Allergy and Infectious Diseases, National Institutes of Health, United States of America

## Abstract

**Background:**

*Staphylococcus aureus* is a versatile bacterial opportunist responsible for a wide spectrum of infections. The severity of these infections is highly variable and depends on multiple parameters including the genome content of the bacterium as well as the condition of the infected host. Clinically and epidemiologically, *S. aureus* shows a particular capacity to survive and adapt to drastic environmental changes including the presence of numerous antimicrobial agents. Mechanisms triggering this adaptation remain largely unknown despite important research efforts. Most studies evaluating gene content have so far neglected to analyze the so-called intergenic regions as well as potential antisense RNA molecules.

**Principal Findings:**

Using high-throughput sequencing technology, we performed an inventory of the whole transcriptome of *S. aureus* strain N315. In addition to the annotated transcription units, we identified more than 195 small transcribed regions, in the chromosome and the plasmid of *S. aureus* strain N315. The coding strand of each transcript was identified and structural analysis enabled classification of all discovered transcripts. RNA purified at four time-points during the growth phase of the bacterium allowed us to define the temporal expression of such transcripts. A selection of 26 transcripts of interest dispersed along the intergenic regions was assessed for expression changes in the presence of various stress conditions including pH, temperature, oxidative shocks and growth in a stringent medium. Most of these transcripts showed expression patterns specific for the defined stress conditions that we tested.

**Conclusions:**

These RNA molecules potentially represent important effectors of *S. aureus* adaptation and more generally could support some of the epidemiological characteristics of the bacterium.

## Introduction

*Staphylococcus aureus* is a ubiquitous Gram-positive bacterium responsible for a wide spectrum of infections ranging from localized to life-threatening diseases. Since the worldwide emergence and spread of methicillin-resistant *S. aureus* (MRSA), this bacterium has revealed a particular capacity to survive and adapt to drastic environmental changes [1]. Until recently *S. aureus* was considered to be the prototype of a nosocomial pathogen but it has now also clearly shown capacity for outbreaks in the community, affecting young and previously healthy people [2]–[4], thus suggesting a major epidemiological evolution. *S. aureus* colonizes approximately 30% of the general population without causing any clinical manifestation [5], [6]. The bacterial genome and its plasticity facilitate the evolution of virulent and drug-resistant strains [7]–[13] in response to major and ever-changing clinical challenges. The microbial strategies also include a unique ability to hide in protected “niches” that are shielded from antimicrobials and host defense components. Several studies have indeed shown that *S. aureus* is able to survive for prolonged periods of time in the cytoplasm of non-phagocytic cells [14], [15]. Thus, the high diversity of diseases caused by *S. aureus* and the breadth of environments colonized by this bacterium strongly relies on the expression regulation of numerous genes, such as virulence and/or metabolism genes. Confronted with these epidemiological changes, there is a marked need to better understand the pathogenicity and virulence of this organism, and to more precisely define the emergence of epidemic clones, as well as their adaptation during the infection phase and their spreading in the hospital or the community. The pathogenicity, virulence or emergence of epidemic clones within MRSA populations is not clearly defined at a genetic level, despite several attempts to identify common molecular features between strains sharing similar epidemiological and/or virulence behavior. These studies included pattern profiling analyses such as MLVA [16], AFLP [17], MLST [18], or microarrays [19]. To date, all approaches failed to unravel direct correlations between the presence of any molecular determinants and clinical outcomes. We hypothesize that bacterial behavior potentially resides in other parts of the genome that were overlooked by previous studies, such as the so-called intergenic regions.

The regulation of the genes encoding virulence factors occurs at multiple levels and in a temporal manner, according to the stress [20], [21]. This suggests that the production of virulence factors is precisely regulated during infection. In this context, RNAs are recognized as major regulators of gene expression [22]–[24]. The RNA regulators, named small RNAs (sRNAs) in prokaryotic organisms, constitute a heterogeneous group of molecules that act by various mechanisms to modulate a wide range of physiological responses. RNAIII, for example, is the largest and best studied sRNA in *S. aureus* that controls the temporal expression of numerous virulence genes encoding exoproteins and cell wall-associated proteins [25]. Overall, sRNAs can modulate transcription, translation, mRNA stability, DNA maintenance or silencing. Thus, these RNA regulators can play a major role in the complex process of epidemicity and virulence as well as for adjusting to diverse environmental stresses. Various regulatory mechanisms involving sRNA have been reported and include changes in RNA conformation using riboswitches, protein binding, base pairing with mRNAs, and interaction with DNA [26], [27].

Despite the importance of sRNAs, their study in the *Staphylococcus aureus* genome is relatively recent, notably due to the lack of appropriate tools to detect them. Most have been detected by *in silico* predictions, such as *spr* and *rsa* small RNAs [26], [28], and their involvement in the regulation of virulence factors or metabolism was then reported [26]. Nowadays, the development of massively parallel analysis methods including high-throughput sequencing (HTS) and tilling arrays allows for detecting such transcripts at the genome-wide level [22], [29], [30]. HTS has the potential to reveal a quasi-exhaustive inventory of the RNAs present in a sample, the dynamic range of the detection sensitivity is only limited by the sequencing depth. HTS thus permits identifying and quantifying the RNA molecules that are expressed at a given time and under a given set of experimental conditions [31], [32].

The aim of our study is therefore to decipher key elements connecting epidemicity of the strains with their genomic content. In other terms, we propose to identify the genomic and transcriptomic features responsible for the “success of *S. aureus*” in the clinical or epidemiological settings. In the present work, we used direct HTS to investigate the *S. aureus* transcriptome for both CDSs and intergenic regions. Fourteen complete genome sequences of *S. aureus* are currently available [33]. We selected strain N315, a fully sequenced and annotated clinical isolate [34], as a model organism because it belongs to the genetic background that was first associated with the glycopeptide-intermediate *S. aureus* (GISA) phenotype. We describe here the first *S. aureus* transcriptional analysis using Illumina-HTS where samples collected at different times of the growth phase were sequenced, oriented and quantified to detect unknown transcripts. This study reveals that approximately 10% of the intergenic regions contain expressed transcripts, indicating that RNA-dependent regulation is certainly far from negligible in this pathogen. In addition, we identified numerous antisense RNA molecules. Overall, these 195 transcripts were classified in eight categories on the basis of their sequence, location and predicted structure yielding to an enriched annotation of N315 genome. We complemented this analysis by RT-qPCR experiments during various stress conditions.

## Results

### Analysis of the Illumina RNA-Seq coverage profiles

In order to analyze the *S. aureus* N315 transcriptome obtained by Illumina-HTS in depth, and particularly to detect the non-annotated regions, we applied the following three criteria to characterize each transcript: (i) detection, (ii) orientation and (iii) expression during *S. aureus* N315 growth in rich medium (MHB). For the two first experiments, RNAs were purified after 4 hours of growth using RNeasy or MirVana isolation kits for comparison purposes. In theory, the MirVana isolation kit provides a procedure allowing an enrichment of RNAs<200 bases. Mapping of the resulting reads to the annotated *S. aureus* N315 genome revealed only marginal quantitative differences between the two types of RNA preparations. We also evaluated the fragmentation methods on ribosomal-free RNA samples by performing a chemical treatment (presence of Zn^2+^ ions) or using a protocol without zinc-fragmentation. Overall, the numbers of reads successfully mapped on the sequences of N315 were similar and reached 95 and 91%, with or without zinc-fragmentation, respectively (Table 1).

**Table 1:** Summary of cDNA samples sequenced by Illumina RNA-Seq and mapped on N315 *S. aureus* genome.

	Detection run	Orientation run		Expression run			
		Zinc-fragmentation	Without Zinc-fragmentation	2h	4h	6h	8h
Mass of RNA used (ng)	300	276	276	172	209	136	95
Read size (nt)	35	32	32	31			
Number of reads	3'528'913	3'936'842	2'585'522	2'391'961	4'100'930	3'203'394	3'474'726
Mapped reads (%)	90	95	91	98	99	99	98

### Mapping of cDNA sequence reads to the annotated *S. aureus* N315 genome

We manually inspected the whole RNA-Seq coverage profile along the genome of *S. aureus* strain N315 and its plasmid to identify all transcription units including non-annotated ones. As expected, the expression profile showed a strong correlation with the positions of previously annotated genes. Among all annotated CDS, 86% displayed a significant coverage (cut-off value: 0.5 average of sequencing coverage), in at least one of the experiments. In addition, the transcriptional profile revealed numerous signals located in intergenic regions, some of which may correspond to potential operons or to 5′- or 3′- untranslated regions of genes.

All detected transcripts are listed in Table S1 and named “Teg”, for “Transcript from Experimental method from Geneva” according to their discovery order. Total RNA sequencing allowed for identification of 150 intergenic transcripts within the 2.8 Mbp of the chromosome and 9 on the 24 kbp of the plasmid of strain N315. These transcripts are not localized to specific regions but show a rather homogenous distribution along the genome and plasmid of strain N315. A total of 9 transcripts (Teg13, 16, 31, 33, 34, 54, 103, 111, 128), not yet annotated in the genome of strain N315, cover small putative ORFs by mapping large regions without any stop codon and some display a particularly unusual length (Table S1). Overall, we detected 160 intergenic transcripts in the genome of N315, encompassing almost 10% of the intergenic regions with relevant size (i.e. larger than the length of reads (Table 1)). The total size of these newly discovered transcripts represents >30'000 bases, i.e. around 4% of the coding sequences annotated in the genome of *S. aureus* strain N315.

**Figure 1:**
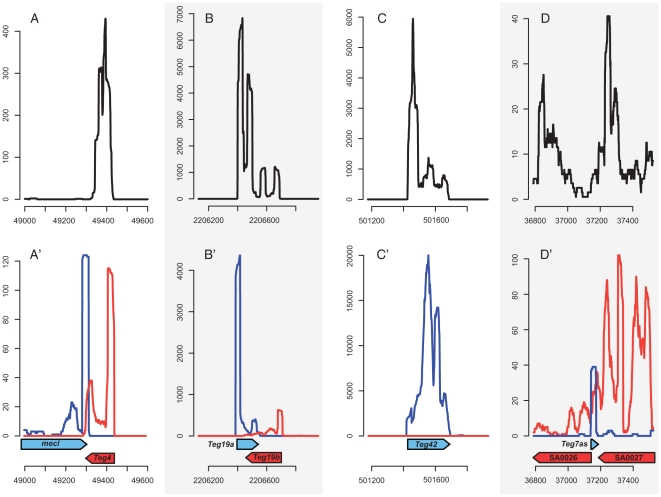
Visualization of different categories of transcripts detected and oriented by HTS RNA-Seq visualized in Artemis genome analyzer browser. The top panels (A–D) display the shape of signals obtained in the *detection* experiment. The lower panels (A′–D′) shows the corresponding signals obtained during the *orientation* experiment. The X-axis corresponds to N315 genomic coordinate and Y-axis symbolizes the normalized coverage. Transcripts expressed on the forward strand are depicted in blue whereas transcripts expressed on the reverse strand appear in red. The vertical axis indicates local sequencing coverage. The arrows represent the position of annotated ORFs deduced from N315 genome sequence and the position of Teg identified in our study. Panels A–C presented various intergenic signals and D shows a typical antisense signal.

Illumina-HTS was then performed with the orientation protocol, after ligation of specific linkers on each strand of the purified RNA preparation. This method allowed us to determine the orientation of all previously identified transcripts in a single run. We validated this experiment by controlling the adequate orientation of the previously annotated transcripts. Strand orientation was unambiguous for all detected transcripts and showed the same read orientation as the reference genome (Figure S1). As only minor differences were observed when using the two dir-mRNA-seq protocols (i.e. with or without zinc fragmentation), we decided to pool these two runs in order to increase the sequencing depth without altering the results. Considering all detected transcripts, the repartition of the transcript coding strands along the circular bacterial genome followed what is known for CDS orientation. Indeed, we observed that the intergenic transcripts localized in the first half of the genome are transcribed by the positive coding strand (21 positive strand transcripts and 15 negative strand transcripts in the first genome quarter; 31 positive strand transcripts and 11 negative strand transcripts in the second genome quarter) whereas the transcripts encoded by the second half of the genome are mostly in the minus stand (12 positive strand transcripts and 28 negative strand transcripts in the third genome quarter; 10 positive strand transcripts and 23 negative strand transcripts in the fourth genome quarter). Some of these transcript profiles are depicted in Figure 1 (A to E) and their corresponding Teg described in Table S1.

### sRNA annotation and classification

**Figure 2:**
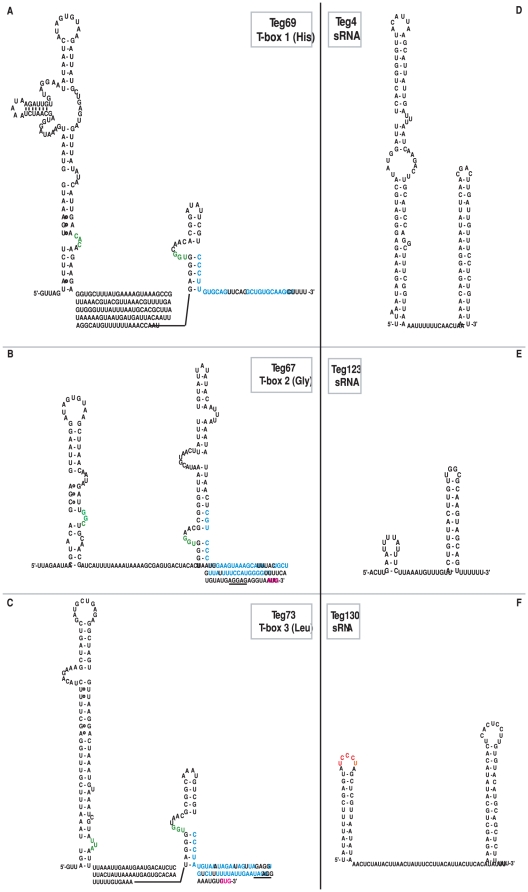
Secondary structures of some riboswitches and *bona fide* small RNAs based on *in silico* analysis. (A, B, C) Examples of the secondary structure of the 5′ untranslated regulatory regions of *S. aureus* mRNAs encoding histidyl-tRNA synthetase (T-box 1), glycine-tRNA synthetase (T-box 2) and leucyl-tRNA synthetase (T-box 3). The models were derived from sequence alignment with the regulatory region of the *Bacillus subtilis* mRNA encoding tyrosyl-tRNA synthetase as derived by Green (2009). Only the specifier hairpin and the expression platform are folded. The T-Box specifier codon are given although the T-box 3 leucine codon is questionable. The predicted residues in the specifier and in the antiterminator loops that participate in pairing with the tRNA are colored in green. The start codon (pink) and the Shine-Dalgarno (SD) binding site (underlined) are represented when located near the end of the T-box. T-box 3 appears to regulate initiation of translation by sequestering the SD binding site of the downstream coding region whereas T-box 1 and T-box 2 regulate the expression of their downstream coding region by a mechanism of termination-antitermination. The structural model of the antiterminator conformation is shown. Residues that participate to the alternate structure (terminator conformation or hairpin sequestering the SD binding site) are colored in blue. (D, E, F) Predicted secondary structure of three *bona fide* small RNAs. The UCCCU sequence motif in red in Teg130 was shown to be conserved in several sRNAs from *S. aureus*. This sequence is well appropriate to bind to the ribosome binding site of target mRNAs [26].

Bioinformatics predictions have been performed to classify the detected transcripts according to their potential regulation mechanism (Table S1). Small RNAs constitute a heterogeneous group of molecules acting by various mechanisms to regulate gene expression. In this context, we have found 14 sRNAs, which correspond to *cis*-regulator riboswitches. Riboswitches are characteristic structures located in the 5′-region of mRNAs that can bind intracellular metabolites. In our study, many classes of such regulators are represented, such as the S-box [35]–[37]. We also identified 17 *cis*-acting regulatory regions that are specifically recognized by a variety of regulatory proteins (r-proteins, GlcT), or by tRNAs (T-box). Otherwise, we have categorized 57 *bona fide* small RNAs which could act in *trans*. Among the small transcripts, 21 sRNAs were detected in the opposite direction of their two flanking genes, and many of them contained a typical Rho-independent transcription terminator hairpin. Thus, these transcripts are most likely synthesized independently of their flanking genes. These observations were used to classify them in the *bona fide* small RNAs group. We have also recognized the stable housekeeping RNAs, such as 4.5S, 6S, RNase P and tmRNA [27], [28]. Evaluation of promoters and terminators, start and stop codons, as well as Shine-Dalgarno sequences allowed us to identify 9 potential CDSs, not yet annotated in RefSeq NC_002745. The annotation software may miss small CDSs and/or CDSs containing alternative start codons. Indeed, five of these nine transcripts (Teg13, 31, 33, 34, 54) show homology with hypothetical protein in others *S. aureus* strains and/or others *Staphylococcus* species, by using Basic Local Alignment Search Tool [38] (BLAST). Finally, 32 signals juxtaposed to one of their flanking genes were classified as 5′- or 3′-UTR of this gene. These regions have been identified and classified in this transcriptomic cartography because of their unusual length for a UTR region. No particular structure or bioinformatics prediction was observed for these 5′/3′ UTR but this observation does not preclude these regions to be involved in regulatory processes. Overall, most of these putative regulatory RNAs present significant secondary structures as exemplified in Figure 2. This analysis unravels conserved regions often showing internal base-pairing that reflects their regulatory function [39]. Note that these sequences are likely of major importance considering their high degree of conservation in the staphylococci (Table S1).

### Antisense RNAs

The orientation protocol allowed us to identify 23 antisense RNAs that showed perfect complementarity with target mRNAs encoded from the same DNA locus (Figure 1F and 1G, for example). Four other transcripts were observed as antisense of the UTR region or antisense of previously identified ncRNAs, such as *sprA*. Interestingly, a recent bioinformatics analysis suggested that type I toxin–antitoxin modules are widely distributed among bacteria [40]. This system consists in small hydrophobic peptides whose expression is repressed by a small antisense RNA. Among these predicted modules, Teg152 and Teg154 are predicted to encode for type I antitoxins in *S. aureus* N315 strain (Table S1). These two ncRNAs are antisense to Teg8 (SprA) and SprG, respectively which are predicted to encode type I toxins [40]. These four RNAs (Teg151, 152, 153, 154) were however classified in the *bona fide* small RNAs group for their intergenic characteristics. In addition, we detected nine antisense RNAs from repeated regions in the genome and three antisense molecules which correspond to previously annotated genes (*bleO*, SA0752, SA0833) but whose 3′ UTR appears as antisense of the flanking gene (Teg39as, 40as, 41as) (Table S1). Most of the antisense RNAs are found in pathogenicity islands. These antisense molecules are putatively acting in at least six functional categories (Clusters of Orthologous Groups; COG) but they display a particularly high frequency in the categories “cell-wall and cell envelopes biogenesis” (M) and “replication, recombination and repair” (L), which are essential biological functions. Another group of *cis*-encoded antisense RNAs may modulate the expression of genes in a potential operon, i.e. between two genes, such as Teg7as (Figure 1F), Teg8as, Teg15as, Teg16as and Teg19as. Based on their location on the mRNA, these transcripts may act as potential inhibitors of translation initiation, as mRNA degradation and/or as mRNA transcription termination.

**Figure 3:**
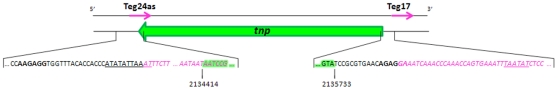
Schematic representation of antisense transcripts in the *tnp* region. Among the transposases annotated in the genome of *S. aureus* N315, antisense RNAs were identified 8 times at the extremities of the *tnp* transcripts, suggesting an important functional role. This example shows Teg24as and Teg17 (pink italic font) and their complementary *tnp* gene (green). In the sequences detailed below, the potential promoter sequences are underlined and the potential Shine-Dalgarno sequences appear in bold fonts.

Antisense transcripts detected in this study correspond typically to *cis*-encoded antisense RNAs, i.e. encoded in the same DNA locus as their complementary targets. Some are predominant in comparison to their antisense gene (Teg8as, 10as, 14as, 15as, 17as, 21as, 22as, 23as, 24as and its duplicated regions, 39as, 40as, 41as), some are less expressed (Teg5as, 6as, 9as, 16as, 19as, 20as, 25as, 26as, 27as, 36as, 37as, 10aspl) and five have almost the same expression levels (Teg7as, 18as, 28as, 38as). Mechanisms of these antisense RNAs may be variable and yield to transcription attenuation, translation inhibition, or mRNA degradation. Several antisense RNAs are complementary to the 3′- end or 5′- end of transposase genes (Figure 3). We were not surprised to find that these transcripts exist in pairs (represented by the same color in Table S1) as they are likely duplicated by the transposase encoded by the associated transposase gene. Potential promoter regions and Shine-Dalgarno sequences have been identified at two locations in the insertion sequence, in the vicinity of antisense transcripts. The genome of N315 contains approximately 25 transposases from 5 major types (for Tn554, IS1181, IS232, or for other poorly characterized IS elements).

Additionally, we have detected 4 antisense RNAs which map to parts of the transcripts identified in intergenic regions. Teg152, Teg153 and Teg154 are complementary to non-coding RNAs previously described by Pichon and Felden [28]. Teg151 has been identified as antisense to the 3′- untranslated region of SA1344 mRNA; we also find that Teg51 is highly expressed in our Illumina-HTS data. These four antisense RNAs have been classified as *bona fide* small RNA, not in the antisense sub-category, due to their intergenic location (see experimental section).

### Orientation of transcripts

The oriented RNA-seq protocol produces reads that are oriented according to the RNA molecules they originate from. The mapping orientation is thus informative, as opposed to regular RNA-seq experiments where the strand information is lost during the cDNA synthesis step. The read mapping procedure revealed that 45% and 55% of the reads map onto the direct and reverse strand of the S. aureus N315 genome, respectively (RefSeq NC_2745). This unequal repartition is due to the highly expressed tRNAs and rRNAs that are more abundant on the reverse strand of the genomic sequence. Housekeeping RNAs are not fully captured during the sample purification procedure and they represent 65% of the total sequenced reads. The remaining 35% of the reads show a mapping repartition of 50.6% and 49.4% for the direct and reverse strand respectively. Investigating the mapping orientation within CDSs revealed that 97% of the reads are oriented according to the coding strands and thus agree with the presence of mRNA transcripts. The remaining 3% correspond either to non-coding RNAs, false-positive read matches or possible gDNA contamination. We observed that reads mapping the non-coding strands are not uniformly spread among the CDSs. To quantify this observation, we computed the non-coding mapping ratio for each CDS displaying a sequencing coverage larger than 10×. This ratio is calculated as *nR*/(*nR*+*nD*), where *nR* and *nD* are the coverage values on the non-coding and coding strand, respectively. Thus, CDSs only covered on the coding strand have a non-coding coverage ratio equal to 0, while higher values reveal the presence of reads that also map the non-coding strand. The CDSs displaying larger non-coding mapping ratios correspond to the antisense sRNAs we manually identified from the coverage signals (details are provided in Table S2).

### Transcript sequence characteristics and conservation

Transcripts detected in our study were compared across phylogeny using the Basic Local Alignment Search Tool [38] (BLAST). All detected transcripts are conserved across all sequenced *S. aureus* strains with a requirement for 80% similarity (Table S1). None of the identified transcripts was unique to strain N315. Some of them are conserved at the bacterial genus, family, or even at the organism class level. Moreover, a few of the RNAs were found to be partially or fully repeated in the genome of *S. aureus* strain N315. These RNAs potentially control important regulatory functions, and this may explain their duplication. These transcripts are listed in Table S1 under “repeated sequences”. Indeed, these duplications in the genome split the assignment of the sequencing coverage for each copy and make it impossible to map a precise expression signal at a defined position.

RFAM allowed us to predict a large majority of the 14 riboswitches and the 17 *cis*-acting regulatory elements found within the bacterial chromosome of strain N315, primarily located in the 5′- untranslated regions (UTR) of mRNAs. Several of these regulatory regions are known to respond to the intracellular concentration of various metabolites (purine, pre-Q, glycine, lysine, Glucosamine-6P, thiamine pyrophosphate, S-adenosyl-methionine, flavin mononucleotide). Furthermore, twelve of the sRNAs contain a putative T-box, and a tandem T-box was found upstream SA1199. Three of them (Teg67, Teg69, Teg73) were not predicted in RFAM. We manually folded the putative T-boxes in order to find their target tRNA (Figure 2A, B, C). It is remarkable that three of these new T-boxes do not contain the consensus signature [41]. The NMR structure of the specifier loop was recently shown to adopt a loop E motif with stacked non canonical basepairs allowing an optimal presentation of the specifier triplet nucleotides for pairing with the anticodon of the sensing tRNA [42]. This specific signature was neither found in Teg67, Teg69 and Teg 73 although alternative base pairing internal loop was predicted for Teg69 with lower reliability. Teg67 harbors a particularly short specifier hairpin containing a Gly codon and a very long antiterminator moiety, but its structure appears quite different from the consensus. Another example is constituted by Teg73 that regulates the expression of leucyl-tRNA synthetase. In that example, the folding of the specifier hairpin that should position a leucine “codon” in an appropriate configuration appears not optimal, raising the question of its functionality (Figure 2A, B, C).

Approximately 10 of these sequences harbor GC content significantly higher than the average GC content of the genome. Among all of these *cis*-acting putative regulatory regions, 14 are transcribed on the positive strand according to RefSeq NC_002745, whereas 17 are transcribed on the minus strand. The genes located downstream of these putative regulatory regions belong to two functional COG categories: “translation, ribosomal structure and biogenesis” and “amino acid transport and metabolism”. Of interest, these regions were found to be highly conserved in sequenced *S. aureus* strains with >96% homology. In addition, significant homology levels, generally ≥80%, were observed in several coagulase-negative staphylococcal species such as *S. haemolyticus*, *S. epidermidis* or *S. saprophyticus* (Table S1). Decreasing the stringency of the homology cut-off allows us to obtain partial homology. BLAST searches using these criteria uncovered the presence of numerous transcripts conserved in *Bacillales* or more frequently in *Firmicutes* (Table S1).

A total of 57 *bona fid*e sRNAs were identified in the bacterial genome on the basis of bioinformatics prediction or based on the literature. Only eight of these sRNAs harbor a GC content that is significantly higher than that of the bacterial chromosome. Similarly, nine sRNAs harbor a GC content significantly lower than that of the genome. In this category, four transcripts show a GC content lower than 25% and reveal partial homology to sequences found in low-GC Gram negative organisms (e.g. *Campylobacter*, *Wolbachia*). Interestingly, most of the previously described transcripts uncovered by bioinformatics strategies were found in this category of sRNAs, such as *rsa* and *spr* [26], [28]. Note also that 26% (n = 14) were observed on documented pathogenicity islands.

Discovered sRNAs are generally in single copy in the genome of strain N315 with the exception of five transcripts that were found in multiple copies (listed as repeated sequences, Table S1). Most of these duplicated transcripts are located in the vicinity of transposases that exist in multiple copies in the genome of strain N315. These transcripts appear to be either species-specific or markers of mobile genetic elements found in other staphylococcal species and are highly conserved (Table S1). Interestingly, one transcript (Teg12) classified in this sub-category shows repeated segments within its primary sequence, similar to STAR (*Staphylococcus aureus* repeats) elements [43]. These transcripts are characterized by short palindromic repeats at regular intervals, leading to a series of loop structures [26].

Functional categories (COG) of genes flanking all small transcripts detected in this study were analyzed and compared to the chromosome content. The repartition of sRNAs is definitely not random but shows particular abundance among specific categories. Small RNAs are flanked by genes of almost all categories but we noticed a particular enrichment for localization between genes involved in carbohydrate and ions metabolism and transport (G and P), translation (J), intracellular trafficking (U) and defense mechanisms (V). Overall, these categories contribute to 25% of the genome content and contain biologically or clinically relevant regions.

Previous studies based on *in silico* prediction, Affymetrix microarrays or sequencing have documented the existence and partially characterized the functions of some sRNAs from *S. aureus* [26], [28], [44]–[47]. Comparison of intergenic sRNAs discovered in these different studies is shown in Table 2. The vast majority of *spr* and *rsa* genes were detected by *in silico* prediction and documented by Northern blots [26], [28]. Only *sprFG2* and *sprA2* are missing from our study in comparison to the work of Pichon and Felden. Similarly, our predicted *rsa* genes are complete with the exception of *rsaB* and *rsaF* which were previously described by Geissmann and colleagues. The works of Roberts [45] and Anderson [44] using Affymetrix microarrays documented the presence of numerous transcripts expressed under various stress conditions that were not found in our study. More recently, a conventional cloning/sequencing approach by Abu-Qatouseh [47] revealed the existence of sRNAs which were differentially expressed in small-colony variants of *S. aureus*. Some of these were not identified in our study. Note that these reports were performed with phylogenetically distant *S. aureus* strains and that the detection cut-offs are probably not comparable between the very different experimental methodologies used in these studies. Finally, our work revealed the presence of some 80 additional intergenic transcripts not reported by any of the previously published studies (Table 2, Table S1).

**Table 2:** Comparison of transcripts detected in intergenic regions in regards to previous analysis.

Study	Discovered transcripts		In common with our study
	*In silico* prediction tested	Experimental validation	
**This paper**		**139**	
**Pichon & Felden 2005 ** [**28**]	**25**	**15**	**14**
	RNAIII, RNaseP, tmRNA, 4.5S, 6S, sprA-G3, IGR59, 330, 1383, 1535A, 1535B, 1537, 1634, 1641c2, 1652, 2069	RNAIII, RNAse P, tmRNA, 4.5S, 6S, sprA-G3	RNaseP/Teg65,tmRNA/Teg150, 4.5S/Teg42, 6S/Teg97, sprA/Teg8, sprA3/Teg153, sprB/Teg9, sprC/Teg10, sprD/Teg14, sprE/Teg15, sprFG/Teg154,sprFG3/Teg19a-19b, IGR59/Teg37, IGR330/Teg145, IGR1634/Teg122
**Roberts 2006 ** [**45**]		**42**	**10**
		WAN01: CBK6-rc_at, CBM1, CBNY and CBNY-rc, CCDF_x, CBPW-rc_s, CBPX-rc_s, CBR1 and CBR1 -rc, CBR5-rc, CBR7 and CBR7-rc, CBR9 and CBR9-rc, CBRA and CBRA-rc, CBRS, CBVV and CBVV-rc, CBVX, CBWS, CBZR-rc, CC05 and CC05-rc, CC7R-rc, CCF1, CCFM-rc, CCGA-rc, 4I74_s, CCHO and CCHO-rc, CCK2 and CCK2-rc, CCK3 and CCK3-rc, CCK4 and CCK4-rc, CCK9, CCM0-rc, CCPS-rc, CBVM, CBVM-rc	WAN01CBPQ_at/Teg42, WAN01CC66_at and WAN01CC66-rc_at/Teg65, WAN01CC8T-rc_at/Teg97, WAN01CCBZ_at/Teg8 and 152, WAN01CCEV_at and WAN01CCEW_x_at and WAN01CCEW-rc_x_at/Teg139, WAN01CCFG-rc_at/Teg154, WAN01CCIL-rc_at/Teg19ab, WAN01CCMM-rc_at/Teg27, WAN01CC2P_at/Teg81, WAN014GIY_at/Teg150
**Anderson 2006 ** [**44**]		**75**	**23**
		S2, S3, S7, S8, S11–15, S17, S19–21, S24, S25, S28, S30, S33–35, S37–40, S43, S45–49, S51–63, S66, S68, S69, S71–75, S79–82, S84, S86, S88, S90, S91, S93, S98–102, S105, S111, S113, S114, S116–118, S121–124	S1/Teg137, S5/Teg110, S6/Teg15, S9/Teg81, S18/Teg64, S23/Teg66, S27/Teg70, S29/Teg74, S32 and S94/Teg139, S36/Teg20, S41/Teg27, S42/Teg29, S44/Teg76, S65/Teg111, S67/Teg84, S70/Teg98, S76/Teg65, S78/Teg97, S85/Teg77, S89/Teg42, S101/Teg90, S107/Teg150, S109/Teg31, S119/Teg154
**Marchais 2009 ** [**46**]	**24**	**7**	**8**
	ND1–17 and RsaOA-OG	RsaOA-OG	RsaOA/Teg144, RsaOB/Teg40, RsaOC/Teg50, RsaOD/Teg67, RsaOE/Teg73, RsaOG/Teg24, ND14/Teg48, ND15/Teg106
**Geissmann 2009 ** [**26**]	**36**	**11**	**22**
	RsaA-K and RsaX01-X25	RsaA-K	RsaA/Teg88, RsaC/Teg90, RsaD/Teg91, RsaE-RsaF/Teg92, RsaG/Teg93, RsaH/Teg94, RsaI/Teg24, RsaJ/Teg96, RsaK/Teg38, RsaX02/Teg118, RsaX03/Teg144, RsaX05/Teg41, RsaX08/Teg50, RsaX11/Teg135, RsaX12/Teg136, RsaX15/Teg137–138, RsaX17/Teg139, RsaX18/Teg27, RsaX20/Teg130, RsaX21/Teg131, RsaX23/Teg153, RsaX25/Teg141
**Abu-Qatouseh 2010 ** [**47**]		**65**	**17**
		Sau: 02,07, 11, 19, 20, 24, 25, 27, 28, 30, 40, 41, 46, 55, 58, 60, 63a, 63b, 64, 69, 76, 81, 82, 85, 5837, 5949, 5960, 5971, 5995, 6041, 6053, 6054, 6057, 6059, 6072, 6128, 6191, 6199, 6229, 6282, 6291, 6318, 6353, 6361, 6387, 6428, 6463, 6469, 6477, 6513, 6526, 6528, 6569, 6590, 6648, 6799, 6817, 6836, 6851, 6889, 6902, 6904, 6930, 6987, 7007	Sau02/Teg102, Sau07/Teg100, Sau11/Teg79, Sau19/Teg131, Sau20/Teg92, Sau24/Teg81, Sau28/Teg82, Sau63b/Teg146, Sau64/Teg88, Sau5837/Teg96, Sau5949/Teg120, Sau5960/Teg104, Sau6053/Teg78, Sau6059/Teg94, Sau6229/Teg57, Sau6428/Teg109, Sau6477/Teg47

The numbers and names of non overlapping transcripts found in intergenic regions are given for each study and compared to the present study. The locus of each transcript has been considered. Three studies [26], [28], [46] used *in silico* prediction in first intention and confirmed the existence of transcripts by experimental validation such as Northern blot or Race-PCR. Only the sRNAs which have been experimentally tested have been taken into account. Otherwise, our study as those of Roberts [45], Anderson [44] and Abu-Qatouseh [47] are based on direct experimental approaches as Affymetrix microarrays, or cloning/sequencing strategy. The number of detected transcripts varies according to the methodologies and the strain used by the author. The study of Geissmann [26] also predicted the known *cis*-acting elements such as the metabolite-sensing riboswitches, the T-box motifs and several protein-mediated antiterminaison events.

As described above, the mapping by HTS does not reach the precision of the RACE-PCR for determining the exact start and end of the expressed RNA. However, the coordinates we assessed in our study showed that the transcript sizes in *S. aureus* N315 are generally around 150 nucleotides (nt) whereas RNAs larger than 300 nt are rare, suggesting that many of them correspond indeed to small RNA regulators, compared to coding sequences showing an average size >1 kbp [34]. In terms of primary sequence characteristics, these different transcripts are heterogeneous considering their respective category, with a median length varying from 285 nt for the antisense RNAs, 108 nt for the 3′ or 5′ UTR, 148 nt for the sRNAs, 141 nt for the riboswitches or 203 nt for the *cis*-acting regulators. Finally, the multicopy transcripts were the smallest molecules with a median size of only 95 nt. The size average of the potential CDS regions is 136 nt (excluding the unclassifiable Teg34).

### mRNA transcript expression during growth-phase by Illumina-HTS

**Figure 4:**
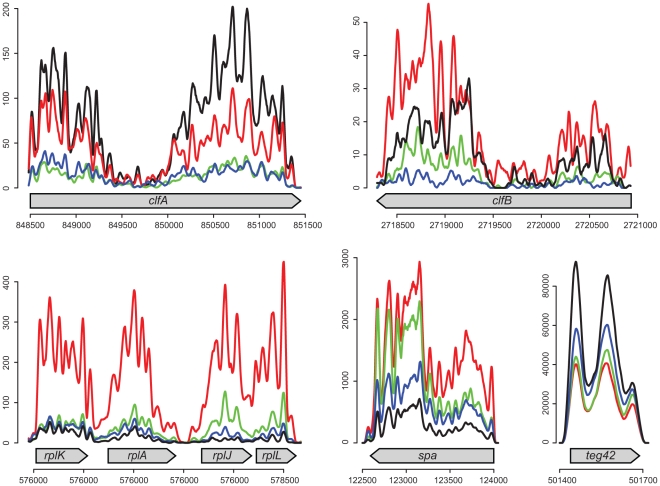
Temporal expression of selected RNAs obtained by RNA-Seq Illumina-HTS. Transcript expression levels are shown at 2 hours (red), 4h (green), 6h (blue), and 8h (black). The X-axis corresponds to N315 genomic coordinate and Y-axis symbolizes the normalized coverage. We smoothed the coverage profiles by using a sliding average window of size 41. The expression profile of control genes has been used for experimental validation. General profiles are consistent with published studies with respect to the temporal expression of these transcripts. The experiment shows that the expression of most small transcripts identified in this study are regulated during the growth phase.

Temporal expression of transcripts at the genome level during the growth-phase was obtained by HTS (Figure 4) whereas precise expression of a subset of 26 sRNAs identified in this study was assessed by RT-qPCR. Expression levels of annotated genes known to contribute to the *S. aureus* pathogenicity were evaluated in the specific context of strain N315 which is *agr*-deficient. Obviously, *hld* was not expressed during the growth kinetics and consequently, RNAIII is missing. The *agr* locus is an important global regulator controlling the temporal expression of several *S. aureus* virulence factors [48]. Nevertheless, strain N315 has an inactive *agr*-system which is not rare in clinical isolates. In fact, N315 is one of the most widespread *S. aureus* lineages responsible for human infections [49], [50]. Expression of virulence factors is also regulated by other partners, acting independently from the *agr*-system. Previous observations regarding sRNA expression suggest that other factors, such as small RNA molecules could also contribute to the regulation mechanisms of virulence factors [28]. A similar absence of expression has been observed in N315 for the whole *ica* operon that contributes to biofilm formation [51] and the *cap* genes encoding for the production of the bacterial capsule [52]. On the other hand, *rpl* or *rps* genes encoding for ribosomal proteins followed previously described kinetics [53]. Bacterial adhesins are important proteins contributing to the attachment to host cells or tissues [54] and the regulation of some of these factors has been previously studied in detail [55]–[58]. In our study, fibronectin-binding proteins A and B were detectable mainly during the early phase of growth, but remained only poorly expressed during the entire growth kinetics in this *agr*-deficient background [57]. Clumping factors A and B which provide the bacterium with the capability to interact with human fibrinogen [59], [60] showed similar expression profiles during the growth-phase. In accordance with previous reports, we noticed high levels of expression of *clfB* at a very early phase of growth [61] which is then followed by an significant decrease. However, in this strain background, *clfB* expression appeared maximal during the later growth times. Finally, the expression of *spa*, the gene encoding the protein which binds the major Fc fragment of immunoglobulins, appeared to be similar to previously published studies [62]. This observation suggests that *agr* acts as an amplifier of *spa* transcript level during the complete growth kinetics. *Agr* is not the driver of *spa* expression, as the synthesis kinetics appear similar irrespective of the presence of an active *agr*. Similar observations are available for other important virulence factors such as *hla* and *hlb* genes encoding for haemolysins, in the absence of a functional *agr*. Based on the mapping obtained from multiple timepoints in our kinetic run (see experimental section), the kinetics of the signals we obtained showed impressive reproducibility, both for the annotated genes and the newly discovered transcripts (Figure 4).

### RT-qPCR validation of HTS data for a subset of sRNA genes expressed during stress

**Figure 5:**
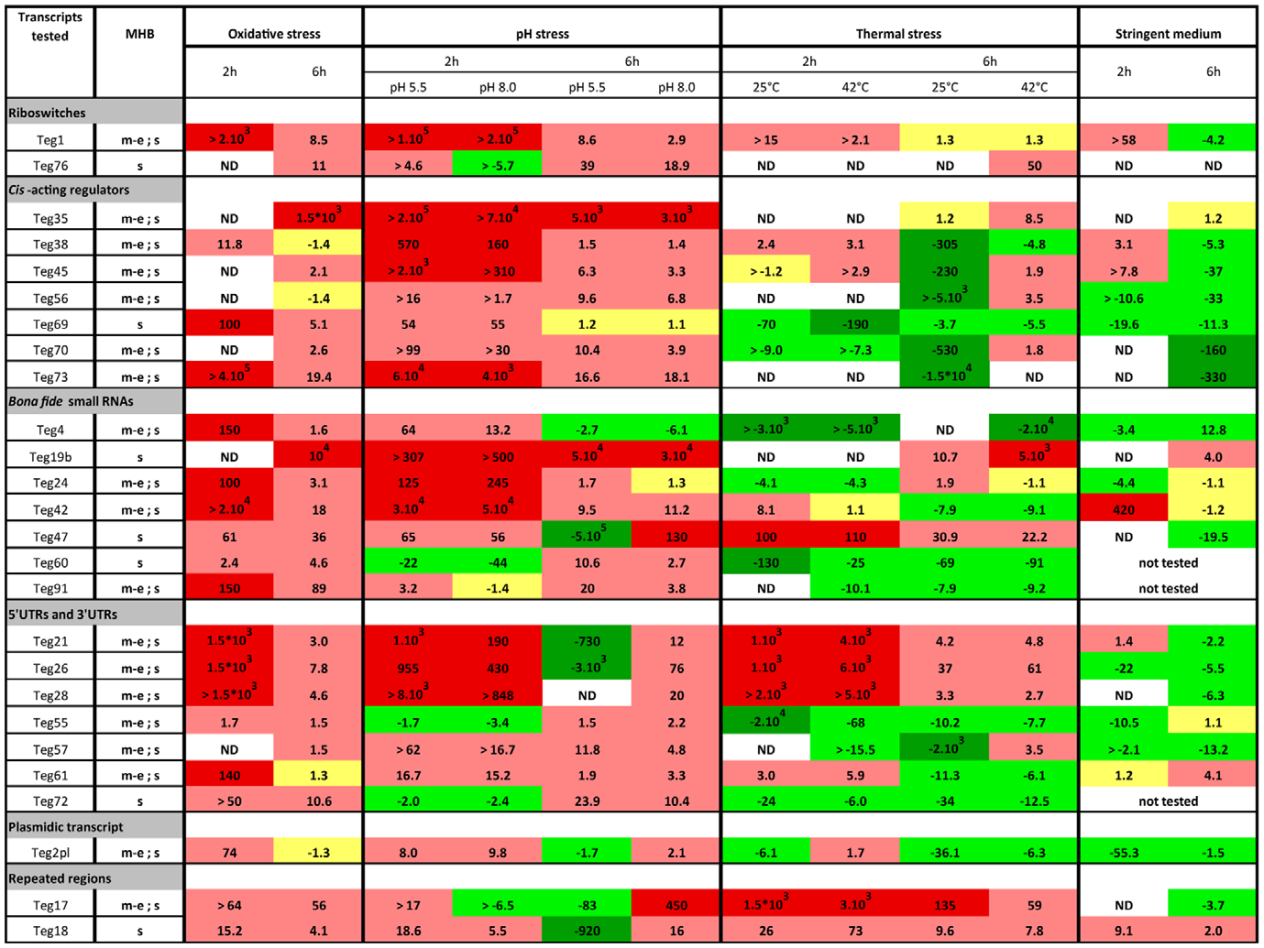
Temporal expression of selected transcripts in rich medium and under various stress conditions. Analysis of the expression of 26 selected transcripts by RT-qPCR in rich medium (MHB) and under four stress conditions: oxidative stress, pH stress, heat and cold shocks, changing of carbon source with stringent medium. The data have been normalized against the *hu* reference gene. We used m-e for mid-exponential phase and s for stationary phase to map the kinetics observed in MHB. Transcript induction or repression has been expressed as fold change against the MHB reference condition. The color scale reflecting the intensity of expression changes: **dark red**: fold change>100, **light red**: 1.3<fold change<100, **yellow**: −1.4<fold change<1.3, **light green**: −100<fold change<−1.4, and **dark green**: fold change>−100. ND corresponds to “Not Determined” value, whenever no expression of tested RNA was visualized before the 40 PCR cycles. The symbol “>” is applied whenever signal was detected for the target but no in the MHB reference. Note that Teg17 and Teg18 are found in multicopy. The expression observed in RT-qPCR is therefore the addition of transcription level from different coding sequences or belongs to another genomic location.

We used RT-qPCR to complete the RNA-Seq expression run with an assessment of the expression of 26 transcripts that are representative of most sRNA sub-categories (Figure 5). The experiments were first performed in rich MHB medium in order to obtain baseline values. Most RNAs are highly expressed in the stationary phase and typically show a significant increase during the transition between the mid-exponential phase and the stationary phase (Figure 5).

Various stress conditions were then tested to mimic environmental changes encountered by the bacteria during the infection, such as pH or temperature variations as well as oxidative or nutrient stresses. Gene expression was evaluated during the early or late exponential phase of growth (2h and 6h). The main results are depicted in Figure 5. In summary, we observe that at 2 hours, thermal, oxidative and pH stresses induce the expression of most of the evaluated transcripts. This induction seems to be particularly important at the two pH values tested during the early (2h) timepoint we observed for several transcripts (Figure 5). On the other hand, during the late-exponential phase of growth, expression variation was systematically lower except for Teg17, Teg47, Teg55, Teg72 and Teg76 (Figure 5). We also found spectacular inductions of expression during the oxidative stress response of 11 small transcripts at 2h (Teg1, 4, 21, 24, 26, 28, 42, 61, 69, 73 and 91). Early responses (2h) to temperature increase or decrease were observed for 8 different transcripts. In contrast, many transcripts were repressed at 6 hours, at both of the tested temperatures (Figure 5). Expression during growth in stringent medium was also remarkable for numerous tested transcripts. At 6h, the expression of the vast majority of our target genes was either not influenced or significantly decreased during growth in minimal medium. Yet some of the tested genes showed increased expression during the early phase of growth (Teg1, 18, 21, 38, 42, 45). Among them, the kinetics of Teg42 (4.5S) illustrates the ubiquitous role of signal recognition particles (SRP), which appear tightly regulated during the growth phase but also during various stress situations such as oxidative or pH shocks as well as nutrient starvation. Note that Teg42 (4.5S) as well as other detected sRNAs appeared particularly abundant (Figure 4) and exposure to stress conditions yielded to impressive fold change values or copy number (Figure 5).

## Discussion

*S. aureus* is an important human pathogen with a spectacular capability to regulate its metabolism in order to survive a variety of changing environmental conditions. Considering the human body as the main replication site for the bacterium, several lines of evidence demonstrate that temperature or pH changes, the presence of reactive oxygen species, nutrient starvation as well as the exposure to various families of antibiotics have an impact on its growth phenotype, but they also unravel a surprising capacity to rapidly adopt behavior allowing survival and persistence. Mechanisms underlying pathogenicity, virulence and the emergence of epidemic clones of *S. aureus*, including MRSA, are not clearly understood yet. Benefitting from HTS capacity, we describe here the largest dataset representing the *S. aureus* transcriptome obtained to date. RNA-Seq allowed us to provide a complete pattern of small RNAs expressed under specific conditions in *S. aureus* strain N315, and to enrich previous bioinformatics approaches, that are currently limited in ability to detect low structured sRNAs.

We identified all transcripts expressed in strain N315 at different time points. This includes the measure of predicted CDS but also the discovery of 35 antisense RNAs and 160 small RNAs expressed from “non-coding” or not (yet) annotated regions. Some of these molecules show unusual consensus sequences that preclude detection by classical algorithms. In the genome of strain N315, we discovered that almost 10% of the expressed transcripts were in fact ignored as of today, permitting us to now properly assess their role in virulence or metabolism. The method used in our study is “observational” and because we know that all detected transcripts were indeed expressed, we know they constitute functional transcription units of the considered organism. Despite their considerable merit, predictive bioinformatics tools or massively parallel methods such as microarrays remain limited by the need for knowledge of consensus or sequence which may not yet exist, and are sometime affected by false-positive [63]–[65]. HTS also allows access to operon visualization, but in this study we focused specifically on the cartography of RNA regulators.

### Riboswitches and *cis*-acting regulators

An important category of regulatory molecules is constituted by riboswitches and *cis*-acting regulators that are responsible for transcription and translation regulation through conformational rearrangement in prokaryotes. This type of *cis*-acting structure was frequently found in the genome of N315 and some known riboswitches [26] were confirmed by our study. The 31 signals identified here revealed particularly conserved structures and to a lesser extent (Figure 2 and Figure S2) revealed that motifs involved in secondary structure formation are conserved within the *Staphylococcus* genus. As it was previously shown for riboswitches and T-boxes, alternative structure may occur. Binding of the effector (metabolite, tRNA) stabilizes one of the two conformers to change the expression of the downstream gene. In many cases, the regulation occurs at the level of transcription termination but a few of these elements also regulate initiation of translation (Figure 2). These riboswitches and *cis*-acting regulators are concentrated within essential metabolic pathways - i.e. mainly those involved in amino acid biosynthesis, but also upstream of regulators involved in the development of biofilms and for adaptation to nutrient starvation [66]. This concentration is not surprising considering that their mode of action consists of interacting with small metabolites to regulate the expression of genes involved in tRNA aminoacylation and in transport and biosynthesis of amino acids [26]. The sequences involved in secondary structures appear highly conserved and share significant homology at the class (*Bacilli*) or the phylum (*Firmicutes*) level. Some mRNA targets of these RNAs have been identified recently in important genes regarding virulence and metabolism [67]. Of interest, a recent work identified a pyrimidine compound binding guanine riboswitches with bactericidal activity against *Staphylococcus aureus* [*68*].

### Antisense RNA molecules

Another category of regulation events consists of antisense RNA mechanisms. Our study revealed a total of 35 antisense sRNAs showing perfect complementarity with annotated gene sequences. This base-pairing ability indicates their roles in the post-transcriptional regulation of gene expression. Their expression is highly variable compared to their cognate mRNA targets, and we are not able to determine the direct relationship between these antisense molecules and their sense mRNA target. We cannot exclude that some of these antisense RNAs act in *trans* on different targets with partial base-pairing, even if the sense complementary RNA appears to be their natural target. Potential silencing of gene expression or alternative processing of the target mRNA, such as sRNA-mRNA duplex degradation, were frequently observed in pathogenicity islands. Note that this category of RNAs was highly conserved at the species level, and some significant portions of these molecules were also found in distant or very distantly-related bacterial species (e.g. *Clostridium* spp. and Gram negative rods), precisely in regions annotated as pathogenicity islands or secretion systems in these organisms. *Cis*-encoded antisense transcripts have been previously found in *Archaea* [69] and in *Bacteriae* [70], with various abundances. Wurtzel and colleagues nicely demonstrated that approximately 8% of the operons in *Sulfolobus solfataricus* are potentially interacting with antisense-specific RNAs. Likewise, the recent study of Abu-Qatouseh [47] in *S. aureus* small colony variants identified 78 RNA candidates transcribed in antisense orientation, representing 55% of detected non-protein coding RNAs. Compared to our findings, this result demonstrated that expression of these antisense RNA molecules is strongly regulated according to the different conditions. A similar predominance for antisense sRNAs was described in cyanobacteria [71]. In humans, antisense RNAs appear to be widespread with estimates that they comprise up to 25% of the transcripts [72], [73]. This number is generally lower in bacteria and particularly in this *S. aureus* N315 strain where we identified this type of organization for only 1.3% of mRNAs, considering our cut-off. Note however that the presence of an antisense RNA in the capsule operon as well as in the staphylococcal secretary antigen (*ssaA*) may have important functions since this envelope structure is critical for the survival of the bacteria in human phagocytic cells [74]. Indeed, SsaA appears highly expressed during the course of an infection and is apparently involved in bacterial susceptibility against specific antibiotic families.

Small RNAs are famous for their capacity to interact with the ribosome binding site of mRNAs encoding transposases and consequently inhibiting their mobility [22], [70], [75]. Interesting structures including antisense RNA molecules were frequently found in the vicinity of transposases in the genome of strain N315. The location of these antisense RNAs (Teg17, 30, 59, 98, 101, 119, 142, 145) at the 5′-UTR of *tnp* suggests their capacity to sequester the transcription and translation initiation domains of *tnp* gene. We hypothesize that small RNAs may be implicated in regulation cascades by their ability to act on transposase gene expression. The interactions are sometimes complex as sRNAs act through transposition action, gene activation, gene inactivation and/or genetic rearrangement [76]. Interestingly, we have identified another group of antisense transcripts of *tnp* genes, which are complementary to the 3′-untranslated region of *tnp* (Teg24as, 29as, 31as, 32as, 33as, 34as, 35as, 30as). Surprisingly, these 3′-antisense RNAs have been localized near the transcription and translation initiation regions of the potential downstream gene. This structural organization suggests that this insertion sequence may activate downstream genes, by the incorporation of initiation sequences upstream of an ORF, and that antisense transcripts could regulate this mechanism (Figure 3). Finally, these processes may contribute to the evolution of the bacterium by affecting the capacity of *S. aureus* to acquire genetic elements implicated in virulence, by facilitating its adaptation to various environments [77] or by acting in *trans* to produce multicopy inhibition [78]. The latter mechanism may be involved in either strict gene regulation or fast evolution of the bacterial genome during exposure to selective pressure such as that encountered during an infection [76]. Recently, this type of antisense sRNA has been documented by Toledo-Arana and colleagues in *Listeria monocytogenes* using tiling arrays [22]. Several RNAs were found partially or fully repeated in strain N315 suggesting important functions related to their duplication and conservation during the evolution process of the organism.

Despite the fact that many fully complementary antisense sRNAs are currently known, the vast majority are found encoded on bacterial plasmids [70], [79]. Many detected antisense RNAs have also been found in IS1181, which was initially identified in a methicillin-resistant *S. aureus* strain [80]. In this study, 10 antisense transcripts were localized in pathogenicity islands and could thus play a key role during *S. aureus* infections. These molecules are present in at least 6 COG categories and are particularly abundant in genes involved in cell wall and cell envelope biogenesis as well as in replication, recombination and repair. We speculate that these transcripts act though base-pairings with their mRNA targets leading to the inhibition of translation initiation, mRNA degradation and/or mRNA transcription termination.

### Small RNAs category

Antisense RNAs recognizing non coding RNA (ncRNAs) were also found in our study. This type of interaction between homologous ncRNAs has been previously described in *Listeria monocytogenes*, another Gram-positive organism, suggesting regulatory networks that involve several sRNAs. The implication of such networks in the colonization and infection processes has been recently documented in that organism [22], [81]. Two of these antisense-sense ncRNAs belong to the type I toxin-antitoxin systems [40] (Table S1).

Our study also reveals that the genome of *S. aureus* strain N315 contains >50 sRNAs located in pathogenicity islands or closely related to key metabolic genes previously identified as virulence genes. Whether these molecules trigger regulatory functions and their respective impact on the virulence of the bacterium remains to be precisely evaluated. Regarding *S. aureus* pathogenesis, the abundance of sRNA in pathogenicity islands (around 30% of the identified sRNA in our study), appears to be of paramount importance. Similar to tRNAs and tmRNA, downstream regions of small RNAs are identified as hotspots for foreign DNA integration [82]. For example, Balbontin have recently discovered that small RNAs could mediate prophage insertion and consequently integration of carried exogenous DNA [83]. This observation could explain the large amount of sRNAs frequently found near virulence genes, which are acquired by horizontal transfer during exposure to high selection pressure occurring, for example, in host infection.

We identified here 57 *bona fide* small RNAs here in specific regions of the bacterial genome such as pathogenicity islands, metabolic genes and flanking transposase genes. For these elements, the GC content was significantly higher than that of the average bacterial chromosome, suggesting their potential acquisition during horizontal gene transfer. Of interest, prediction of the secondary structure reveals that many of these sRNAs are characterized by several stable hairpin motifs, and 30% of them carry an unpaired UCCC motif. This motif has been recently shown to be a functional sequence that is used to target the ribosome binding sites of target mRNAs such as RNAIII [84] and RsaE [26]. One of these typical sRNAs is shown in Figure 2F.

The housekeeping sRNA molecule, the major constituent of the signal recognition particle (SRP), was identified in *S. aureus* [28]. In bacteria, SRP delivers proteins to the plasma membrane or participates in post-translational protein sorting [85]. SRP is ubiquitous and found in all living organisms, but its size and the characteristics that lead to secondary structures vary between the different phylogenetic groups. In *S. aureus*, the main loop of SRP sRNA involves 35 perfectly paired nucleotides. The sequence of this loop appears extremely conserved within *Staphylococcaceae* SRP sRNAs, and also well conserved in the *Firmicutes* phylum, indicating a common evolution which explains the conservation of the loop structure. This suggests a common ancestor preceding the divergence to lower levels of the phylogenic tree, mainly accumulating punctual mutations in the non-paired regions. Expression of sRNAs involved in SRP formation is highly influenced by environmental conditions, including medium and physico-chemical composition [26] (See Teg42 in Figure 4 and Figure 5). In that sense, stress conditions have major impacts on the expression of the sRNA discovered in our study (Figure 5). Note also that the expression study performed by RT-qPCR is representative of a large panel of RNA categories such as riboswitches (2/26 tested transcripts), *cis*-acting regulators (7/26), *bona fide* small RNAs (7/26), UTR regions (7/26), repeated sequences (2/26) and plasmid RNAs (1/26). Temperature as well as pH values, presence of oxidative stress and growth in minimal media allowed the identification of sRNAs specifically responsive to particular stress conditions. We conclude that stress conditions have a major impact on the transcript expression and therefore confirm that these RNAs could be implicated in gene regulation and bacterial adaptation to drastic environmental changes.

Our study is affected by potential technical limitations related to the utilization of i) enzymatic steps that could bias the sequence representation, ii) arbitrarily–defined cut-offs for the detection of transcripts. This large inventory, the largest to date, will likely increase when further experiments are performed in other growth conditions or using different *S. aureus* strains. This study already represents an important milestone in the characterization of the *S. aureus* transcriptome and genome, and provides a foundation for studies aimed at elucidating the biology and virulence of this ubiquitous human pathogen.

### Conclusions

In the genome of *S. aureus* strain N315, we identified 160 small RNA molecules in regions formerly considered to be intergenic, and an additional 35 that were detected for their antisense properties against cognate CDS. Some of the small RNAs are localized in biologically or clinically relevant regions, between key metabolic or virulence genes and especially within pathogenicity islands as well as within the methicillin-resistance element. Most of them are temporally regulated during growth phase and affected by various stress conditions, suggesting their implication in the adaptability of this ubiquitous human pathogen. The sizeable amount of data generated by Illumina-HTS and their analysis contributes a broad repository for future investigations of sRNAs. We expect that a systematic analysis of such sRNA networks will shed new light on the virulence and epidemiology of *S. aureus*.

## Materials and Methods

### Bacterial strain, culture conditions and RNA preparation

*S. aureus* strain N315 was grown in fresh Mueller-Hinton Broth (MHB) at 37°C, after a 1∶100 dilution of overnight cultures. For kinetics analysis, total RNA was purified at 2h, 4h, 6h and 8h of growth as previously described [15]. Total RNA of N315 was extracted with the RNeasy (Qiagen) or the MirVana isolation kit (Ambion). For HTS experiments, total RNA was treated with the MICROBExpress kit (Ambion) to limit contamination by multicopy structural rRNAs, following manufacturer's instructions with slight modifications. The maximal amount of total RNA per tube was limited to 4–5 µg because previous experiments showed important contamination levels when using 10 µg. Between each purification step, RNA quality and quantity were determined by Bioanalyzer (Agilent) using the RNA Nano chips, and quantified using the ND-8000 (NanoDrop Technologies).

For the evaluation of transcript expression by quantitative RT-PCR, *S. aureus* strain N315 was exposed to various stress conditions. Cultures were performed in the presence of 1 mM of paraquat (Sigma) for 30 minutes, or grown in the stringent medium NZM (10 g/l casein tryptone digested, Fluka 95039; 5 g/l NaCl; 2 g/l MgSO_4_–7H_2_0; pH 7.0). For thermal shocks, bacterial cells were subjected to either a cold (25°C) or a heat shock (42°C) during 1 hour after exponential growth. For the pH stress, cells were centrifuged 5 min at 5,000 g and resuspended in fresh MHB media buffered at pH 5.5 or pH 8.0 during 30 min. The cells were then rapidly centrifuged and stabilized in acetone/ethanol (1/1) [15]. Total RNA was extracted with CellsDirect One-Step RT-qPCR kit (Invitrogen) including an extensive DNAse treatment (10× final concentration, as suggested by the manufacturer) for the analysis by quantitative RT-PCR.

### Library construction and Illumina RNA-seq runs

We performed three different Illumina RNA-seq experiments; i) a detection, ii) an orientation and iii) a kinetic run. The detection experiment was aim at revealing intergenic sRNAs. The orientation experiment aimed at showing the strand of transcription for all detected sRNAs and at identifying antisense RNAs. The kinetic experiment consisted in a set of four RNA-seq runs to map the transcriptome at 2h, 4h, 6h, and 8h of growth in MHB.

Briefly, the detection run was performed on double-stranded cDNAs prepared from purified DNAse-treated transcripts (MicrobExpress, Ambion) using random primers and RNAse H. The double stranded cDNAs were fragmented by nebulization. After ends-repair and purification, the fragments were ligated with Illumina genomic double-stranded adapters. After size-selection on agarose gel to recover fragments with inserts of approx. 30–150 bp, the library was amplified by PCR with Phusion polymerase. PCR cycling conditions were: i) enzyme activation: 98°C for 30 seconds, ii) denaturation: 98°C for 10 seconds, iii) annealing: 60°C for 30 seconds, iv) elongation: 72°C for 30 seconds, and v) final extension: 72°C for 5 seconds. Steps 2, 3 and 4 were repeated 17 times. The library was then sequenced on the Illumina Genome Analyzer GA for 36 cycles.

The orientation run was performed on transcripts previously purified by the MirVana isolation kit (Ambion) and prepared using a dir-mRNA-SEQ protocol starting either from fragmentation with zinc or from non-fragmented transcripts. After ends-reparation, RNAs were ligated with single-stranded small RNA adapters to preserve the strand orientation. The 3′-adapter was ligated first, followed by the bar-coded 5′-adapter. After cDNA synthesis and PCR amplification for 15 cycles, the library was size-selected on agarose gel to recover fragments with inserts of 15–100 bp.

The kinetic run was performed on purified transcripts (MirVana isolation kit) following fragmentation with zinc using illumina's mRNA-SEQ kit. Double-stranded cDNA fragments were prepared with random primers and RNaseH. After ends-reparation and purification, fragments were ligated with Fasteris-designed bar-coded genomic adapters. After size-selection on agarose gel to recover fragments with inserts in the range of 100–200 bp, the library was amplified by PCR for 18 cycles. The libraries were then sequenced in one channel in the Illumina genome Analyzer GAII for 36 cycles.

### Read mapping and visualization

The standard procedure for transcriptome analysis consists in mapping the reads onto the genomic sequence. For this purpose, we used the MAQ software [86] with default parameters for mapping the sequenced reads onto the *Staphylococcus aureus* strain N315 [34], considering both, the genomic and plasmid sequences (RefSeq NC_2745 and NC_3140). This mapping allowed computing a sequencing coverage by counting the number of time each base of the genome was covered by a sequence read. This coverage profile reflects the transcriptional activity across the genome. For visualization and analysis of this coverage, we used the Artemis genome viewer [87] that allows studying the RNAs expression in the context of the annotated genes. For the orientation experiment, we computed separately the coverage profiles corresponding to the reads mapped onto the direct strand from the ones mapped onto the reverse complement strand of the reference sequence.

### Quantification by high-throughput sequencing

Total RNA of N315 was extracted and purified as described above at 2, 4, 6 and 8h. Individual RNA preparations were fragmented and ligated with specific tags before reverse-transcription and ends-repair steps. Similar amounts of RNA from these 4 conditions were mixed and sequenced using Illumina. Simple normalization was applied following coverage obtained for the individual time points.

### Quantitative real-time PCR (RT-qPCR) from RNA

Transcript-specific primers were developed for 26 intergenic transcripts and for the *hu* internal control gene [88] using the Vector NTI Advance™ 10 software (Invitrogen). Sequences and optimized concentrations of all primers used for these quantifications are shown in Table S3. *S. aureus* N315 total RNAs were amplified and quantified by using a one-step SYBR® Green Quantitative RT-PCR Kit (Sigma) in a final volume of 15 µl. The reactions were performed in a StepOne Real-Time PCR device (Applied Biosystems). For these experiments, we used a MMLV-RT (1U/µl), SYBR Green Taq Ready Mix for RT-qPCR, and primers according to the concentrations mentioned in the Table S3. ROX was used for fluorescence normalization. Two µl of RNA CellsDirect extracts for the 26 transcripts of interest were added whereas 2 µl of a 1∶1,000 dilution of the same sample was used for *hu* quantification. Results are average of two independent determinations using the following RT-PCR cycling conditions: i) reverse transcription: 42°C for 30 minutes, ii) enzyme activation: 94°C for 2 minutes, iii) cDNA denaturation: 94°C for 15 seconds, and iv) annealing and elongation: 60°C for 60 seconds. Steps 3–4 were repeated 40 times. Differences in Ct values between tested transcripts and *hu* signals were used for normalization purpose and based on the MHB medium condition at 2h as a reference. The fold change was expressed as the inverse exponential of the difference between MHB Ct (reference) and the stress condition Ct.

### Bioinformatics analysis of intergenic and antisense small RNA transcripts

All the small RNA transcripts detected in intergenic regions by the mapping of all transcripts in the Artemis annotation environment were analyzed with regard to their location, their characteristics, their potential function and their previous identification in the literature. For each intergenic small RNA transcript, several analyses were performed. We identified the presence of a putative related ribosome binding site and/or a promoter at the 5′-end. We checked the 3′-end for the presence of a rho-independent terminator of transcription. The orientation of the neighbor genes was also considered to classify small RNA transcripts. Five classes of small RNA transcripts were therefore defined that included: putative small CDSs, *cis*-acting regulatory elements, riboswitches, *bona fide* small RNAs and antisense RNAs. We computed the GC content of each small RNA transcript, considering that the presence of a bias in the GC content would be an argument in favor of *bona fide* small RNA as it is the case for tRNA and rRNA in that species. The presence of such a bias has been evaluated by considering two standard deviations of the lower tRNA GC percentage in comparison with the average GC percentage of the *S. aureus* N315 genome (32.5%). The cut-off for such a higher GC percentage was set to 42%. For each category, we scanned the transcript for the presence of a UCCC motif as described previously [26]. All small RNA transcripts were also compared to those identified by the five previously published studies [26], [28], [44]–[46]. In that case, only the locus was considered for all overlapping identified regions (not the strand orientation). Data available in the study of Anderson and colleagues were mapped to the N315 genomic sequence by using the Basic Local Alignment Tool [38] with default parameters. The sequence of each small RNA transcript was enriched by annotation data whenever possible by considering both RFAM and Genbank databanks and the literature for *sprA–G* sRNAs [28] and other stable *rsa* RNAs, [26]. NCBI and RFAM databases provided more than 110 annotated RNAs for the *S. aureus* N315 genome including essentially tRNAs and rRNAs at NCBI while other putative sRNAs and riboswitches were found annotated in the RFAM database. For all the transcripts that lacked an annotation, a comparative analysis was performed by using BLAST with default parameters. This analysis was completed by both the HMM approach previously described by Geissmann [26] to identify GC-rich regions and RNasim comparative analysis [26] that included Wu-blast 2.0 pairwise comparisons of sequences for searching similarities, and QRNA [89] to identify base substitution patterns in pairwise alignments that could correspond to a conserved RNA secondary structure. The interpretation of our data was facilitated by the ApolloRNA program, which was used to assign and visualize all the available information on the annotated *S. aureus* N315 genome [90]. Structure prediction was performed as previously described [26].

## Supporting Information

Figure S1 Reads mapping onto the *S. aureus* genomic sequence from dir-mRNA-SEQ protocol.
(1.51 MB TIF)

Figure S2 Secondary structures of some sRNAs *bona fide* based on *in silico* analysis.
(0.14 MB DOC)

Table S1 List of transcripts discovered by Illumina-HTS. Categories and identification of all transcripts discovered by RNA-Seq Illumina-HTS in intergenic regions or in antisense (annotated as “as”) of previously annotated genes in *S. aureus* N315. Some of these transcripts are found in multiple copies, preventing precise quantification of the signal in each of the multiple locations. The bioinformatics predictions allowed us to classify the intergenic signals in various categories such as riboswitches, *bona fide* small RNAs, 5′ or 3′ units of transcription and small CDSs. Transcripts of plasmid origin are also reported and annotated as “pl”. a This method allows to map the approximate transcriptional start and end points of the small RNAs. Based on characterized CDS with carefully mapped origins, we evaluated the precision of this method at +20 nt. b Coding strand symbol around represent flanking CDSs. The middle one indicates the orientation of the small transcripts. c Sequence conservation among publicly available bacterial sequences realized by BLAST. The strict sequence conservation was evaluated with a cut-off value of 80% on the sequence totality. The nomenclature indicates the conservation level such as *S. aureus* specie (sp), *Staphylococcus* genus (g), *Bacillales* order (o), *Bacilli* class (c), *Firmicutes* phylum (p) or Bacteria kingdom (k). Partial homology was also considered with a cut-off value of 90% of homology on >1/3 of the entire sequence. We have considered that *S. caseolyticus*, otherwise known as *Macrococcus caseolyticus*, as belonging to the *Staphylococcus* genus [91]. d HMM analysis and the sequence conservation among *S. aureus*, staphylococcal species, and firmicutes (CA) were previously performed [26]. e Transcripts in the same box have the same sequence found at multiple locations within the bacterial genome. Signals showing the same color appear closely related: the transposases antisense signal is always followed by another transcript in the 5′-UTR of the same transposase.
(0.10 MB XLS)

Table S2 Sequencing coverage of the coding and non-coding strands in the individual CDSs. The oriented RNA-seq protocol allows differentiating direct and reverse sequencing coverage. Thus, the reads coverage in the individual CDSs can be separated into coding and non-coding coverage. This table enumerates all CDSs of *Staphylococcus aureus* strain N315 having a coverage value of at least 10×. Columns 2 and 3 show, for each CDS, the coverage values for the coding and non-coding strand, respectively. The table is sorted according to the percentage of non-coding coverage (column 4). The CDSs displaying a high percentage of non-coding coverage are good candidates for being regulated by an antisense sRNA. Column 11 shows the corresponding Teg, whenever it has been manually identified from the transcriptomic profile (see Table S1). Some CDSs were not covered enough to allow a clear and reliable identification of a RNA signal from the transcriptional profile. However, the high number of CDSs displaying a significant level of non-coding coverage suggests the antisense RNAs to be an important regulation mechanism in the bacteria.
(0.27 MB XLS)

Table S3 Characteristics of oligonucleotides used in the RT-qPCR assay.
(0.06 MB DOC)

## References

[1] Boyce JM (1992). Methicillin-resistant *Staphylococcus aureus* in hospitals and long-term care facilities: microbiology, epidemiology, and preventive measures.. Infect Control Hosp Epidemiol.

[2] Campbell KM, Vaughn AF, Russell KL, Smith B, Jimenez DL (2004). Risk factors for community-associated methicillin-resistant *Staphylococcus aureus* infections in an outbreak of disease among military trainees in San Diego, California, in 2002.. J Clin Microbiol.

[3] Jones ME, Mayfield DC, Thornsberry C, Karlowsky JA, Sahm DF (2002). Prevalence of oxacillin resistance in *Staphylococcus aureus* among inpatients and outpatients in the United States during 2000.. Antimicrob Agents Chemother.

[4] Kazakova SV, Hageman JC, Matava M, Srinivasan A, Phelan L (2005). A clone of methicillin-resistant *Staphylococcus aureus* among professional football players.. N Engl J Med.

[5] Waldvogel FA, Bisno AL (2000). Infections associated with indwelling medical devices.

[6] Archer GL (1998). *Staphylococcus aureus*: a well-armed pathogen.. Clin Infect Dis.

[7] Hiramatsu K, Aritaka N, Hanaki H, Kawasaki S, Hosoda Y (1997). Dissemination in Japanese hospitals of strains of *Staphylococcus aureus* heterogeneously resistant to vancomycin.. Lancet.

[8] Hiramatsu K, Okuma K, Ma XX, Yamamoto M, Hori S (2002). New trends in *Staphylococcus aureus* infections: glycopeptide resistance in hospital and methicillin resistance in the community.. Curr Opin Infect Dis.

[9] Appelbaum PC (2006). MRSA–the tip of the iceberg.. Clin Microbiol Infect.

[10] Borg MA, Zerafa R, Morrison D, Cuschieri P (2005). Incidence of glycopeptide hetero-intermediate *Staphylococcus aureus* strains in Maltese hospitals.. Clin Microbiol Infect.

[11] Ferraz V, Duse AG, Kassel M, Black AD, Ito T (2000). Vancomycin-resistant *Staphylococcus aureus* occurs in South Africa.. S Afr Med J.

[12] Lulitanond A, Engchanil C, Chaimanee P, Vorachit M, Ito T (2009). The first vancomycin-intermediate *Staphylococcus aureus* strains isolated from patients in Thailand.. J Clin Microbiol.

[13] Sng LH, Koh TH, Wang GC, Hsu LY, Kapi M (2005). Heterogeneous vancomycin-resistant *Staphylococcus aureus* (hetero-VISA) in Singapore.. Int J Antimicrob Agents.

[14] Bayles KW, Wesson CA, Liou LE, Fox LK, Bohach GA (1998). Intracellular *Staphylococcus aureus* escapes the endosome and induces apoptosis in epithelial cells.. Infect Immun.

[15] Garzoni C, Francois P, Huyghe A, Couzinet S, Tapparel C (2007). A global view of *Staphylococcus aureus* whole genome expression upon internalization in human epithelial cells.. BMC Genomics.

[16] Sabat A, Krzyszton-Russjan J, Strzalka W, Filipek R, Kosowska K (2003). New method for typing *Staphylococcus aureus* strains: multiple-locus variable-number tandem repeat analysis of polymorphism and genetic relationships of clinical isolates.. J Clin Microbiol.

[17] Melles DC, Gorkink RF, Boelens HA, Snijders SV, Peeters JK (2004). Natural population dynamics and expansion of pathogenic clones of *Staphylococcus aureus*.. J Clin Invest.

[18] Enright MC, Spratt BG (1999). Multilocus sequence typing.. Trends Microbiol.

[19] Lindsay JA, Moore CE, Day NP, Peacock SJ, Witney AA (2006). Microarrays reveal that each of the ten dominant lineages of *Staphylococcus aureus* has a unique combination of surface-associated and regulatory genes.. J Bacteriol.

[20] Novick RP, Geisinger E (2008). Quorum sensing in staphylococci.. Annu Rev Genet.

[21] Somerville GA, Proctor RA (2009). At the crossroads of bacterial metabolism and virulence factor synthesis in Staphylococci.. Microbiol Mol Biol Rev.

[22] Toledo-Arana A, Dussurget O, Nikitas G, Sesto N, Guet-Revillet H (2009). The *Listeria* transcriptional landscape from saprophytism to virulence.. Nature.

[23] Romby P, Charpentier E (2010). An overview of RNAs with regulatory functions in gram-positive bacteria.. Cell Mol Life Sci.

[24] Toledo-Arana A, Repoila F, Cossart P (2007). Small noncoding RNAs controlling pathogenesis.. Curr Opin Microbiol.

[25] Novick RP, Ross HF, Projan SJ, Kornblum J, Kreiswirth B (1993). Synthesis of staphylococcal virulence factors is controlled by a regulatory RNA molecule.. EMBO J.

[26] Geissmann T, Chevalier C, Cros MJ, Boisset S, Fechter P (2009). A search for small noncoding RNAs in *Staphylococcus aureus* reveals a conserved sequence motif for regulation.. Nucleic Acids Res.

[27] Waters LS, Storz G (2009). Regulatory RNAs in bacteria.. Cell.

[28] Pichon C, Felden B (2005). Small RNA genes expressed from *Staphylococcus aureus* genomic and pathogenicity islands with specific expression among pathogenic strains.. Proc Natl Acad Sci U S A.

[29] Perkins TT, Kingsley RA, Fookes MC, Gardner PP, James KD (2009). A strand-specific RNA-Seq analysis of the transcriptome of the typhoid bacillus *Salmonella typhi*.. PLoS Genet.

[30] Yoder-Himes DR, Chain PS, Zhu Y, Wurtzel O, Rubin EM (2009). Mapping the *Burkholderia cenocepacia* niche response via high-throughput sequencing.. Proc Natl Acad Sci U S A.

[31] MacLean D, Jones JD, Studholme DJ (2009). Application of ‘next-generation’ sequencing technologies to microbial genetics.. Nat Rev Microbiol.

[32] Beaume M, Hernandez D, Francois P, Schrenzel J (2009). New approaches for functional genomic studies in staphylococci.. Int J Med Microbiol.

[33] NCBI (2009). http://www.ncbi.nlm.nih.gov/genomes/lproks.cgi.

[34] Kuroda M, Ohta T, Uchiyama I, Baba T, Yuzawa H (2001). Whole genome sequencing of meticillin-resistant *Staphylococcus aureus*.. Lancet.

[35] Green NJ, Grundy FJ, Henkin TM (2010). The T box mechanism: tRNA as a regulatory molecule.. FEBS Lett.

[36] Roth A, Breaker RR (2009). The structural and functional diversity of metabolite-binding riboswitches.. Annu Rev Biochem.

[37] Dambach MD, Winkler WC (2009). Expanding roles for metabolite-sensing regulatory RNAs.. Curr Opin Microbiol.

[38] Altschul SF, Gish W, Miller W, Myers EW, Lipman DJ (1990). Basic local alignment search tool.. J Mol Biol.

[39] Vogel J (2009). A rough guide to the non-coding RNA world of *Salmonella*.. Mol Microbiol.

[40] Fozo EM, Makarova KS, Shabalina SA, Yutin N, Koonin EV (2010). Abundance of type I toxin-antitoxin systems in bacteria: searches for new candidates and discovery of novel families.. Nucleic Acids Res.

[41] Vitreschak AG, Mironov AA, Lyubetsky VA, Gelfand MS (2008). Comparative genomic analysis of T-box regulatory systems in bacteria.. RNA.

[42] Wang J, Henkin TM, Nikonowicz EP (2010). NMR structure and dynamics of the Specifier Loop domain from the *Bacillus subtilis* tyrS T box leader RNA.. Nucleic Acids Res.

[43] Cramton SE, Schnell NF, Gotz F, Bruckner R (2000). Identification of a new repetitive element in *Staphylococcus aureus*.. Infect Immun.

[44] Anderson KL, Roberts C, Disz T, Vonstein V, Hwang K (2006). Characterization of the *Staphylococcus aureus* heat shock, cold shock, stringent, and SOS responses and their effects on log-phase mRNA turnover.. J Bacteriol.

[45] Roberts C, Anderson KL, Murphy E, Projan SJ, Mounts W (2006). Characterizing the effect of the *Staphylococcus aureus* virulence factor regulator, SarA, on log-phase mRNA half-lives.. J Bacteriol.

[46] Marchais A, Naville M, Bohn C, Bouloc P, Gautheret D (2009). Single-pass classification of all noncoding sequences in a bacterial genome using phylogenetic profiles.. Genome Res.

[47] Abu-Qatouseh LF, Chinni SV, Seggewiss J, Proctor RA, Brosius J (2010). Identification of differentially expressed small non-protein-coding RNAs in *Staphylococcus aureus* displaying both the normal and the small-colony variant phenotype.. J Mol Med.

[48] Novick RP (2003). Autoinduction and signal transduction in the regulation of staphylococcal virulence.. Mol Microbiol.

[49] Sakoulas G, Eliopoulos GM, Fowler VG, Moellering RC, Novick RP (2005). Reduced susceptibility of *Staphylococcus aureus* to vancomycin and platelet microbicidal protein correlates with defective autolysis and loss of accessory gene regulator (*agr*) function.. Antimicrob Agents Chemother.

[50] Sakoulas G, Eliopoulos GM, Moellering RC, Wennersten C, Venkataraman L (2002). Accessory gene regulator (*agr*) locus in geographically diverse *Staphylococcus aureus* isolates with reduced susceptibility to vancomycin.. Antimicrob Agents Chemother.

[51] Cramton SE, Gerke C, Schnell NF, Nichols WW, Gotz F (1999). The intercellular adhesion (*ica*) locus is present in *Staphylococcus aureus* and is required for biofilm formation.. Infect Immun.

[52] Mckenney D, Pouliot KL, Wang Y, Murthy V, Ulrich M (1999). Broadly protective vaccine for *Staphylococcus aureus* based on an in vivo-expressed antigen.. Science.

[53] Corbin RW, Paliy O, Yang F, Shabanowitz J, Platt M (2003). Toward a protein profile of *Escherichia coli*: comparison to its transcription profile.. Proc Natl Acad Sci U S A.

[54] Foster TJ, Hook M (1998). Surface protein adhesins of *Staphylococcus aureus*.. Trends Microbiol.

[55] Cheung AL, Koomey JM, Butler CA, Projan SJ, Fischetti VA (1992). Regulation of exoprotein expression in *Staphylococcus aureus* by a locus (*sar*) distinct from *agr*.. Proc Natl Acad Sci U S A.

[56] Cheung AL, Ying P (1994). Regulation of alpha- and beta-hemolysins by the sar locus of *Staphylococcus aureus*.. J Bacteriol.

[57] Xiong YQ, Bayer AS, Yeaman MR, Van Wamel W, Manna AC (2004). Impacts of *sarA* and *agr* in *Staphylococcus aureus* strain Newman on fibronectin-binding protein A gene expression and fibronectin adherence capacity in vitro and in experimental infective endocarditis.. Infect Immun.

[58] Entenza JM, Moreillon P, Senn MM, Kormanec J, Dunman PM (2005). Role of sigmaB in the expression of *Staphylococcus aureus* cell wall adhesins ClfA and FnbA and contribution to infectivity in a rat model of experimental endocarditis.. Infect Immun.

[59] McDevitt D, Francois P, Vaudaux P, Foster TJ (1994). Molecular characterization of the clumping factor (fibrinogen receptor) of *Staphylococcus aureus*.. Mol Microbiol.

[60] Ni ED, Perkins S, Francois P, Vaudaux P, Hook M (1998). Clumping factor B (ClfB), a new surface-located fibrinogen-binding adhesin of *Staphylococcus aureus*.. Mol Microbiol.

[61] McAleese FM, Walsh EJ, Sieprawska M, Potempa J, Foster TJ (2001). Loss of clumping factor B fibrinogen binding activity by *Staphylococcus aureus* involves cessation of transcription, shedding and cleavage by metalloprotease.. J Biol Chem.

[62] Vandenesch F, Kornblum J, Novick RP (1991). A temporal signal, independent of *agr*, is required for *hla* but not *spa* transcription in *Staphylococcus aureus*.. J Bacteriol.

[63] Puskas LG, Zvara A, Hackler L, Van Hummelen P (2002). RNA amplification results in reproducible microarray data with slight ratio bias.. Biotechniques.

[64] Reyes-Lopez MA, Mendez-Tenorio A, Maldonado-Rodriguez R, Doktycz MJ, Fleming JT (2003). Fingerprinting of prokaryotic 16S rRNA genes using oligodeoxyribonucleotide microarrays and virtual hybridization.. Nucleic Acids Res.

[65] Gordon JJ, Towsey MW, Hogan JM, Mathews SA, Timms P (2006). Improved prediction of bacterial transcription start sites.. Bioinformatics.

[66] Pohl K, Francois P, Stenz L, Schlink F, Geiger T (2009). CodY in *Staphylococcus aureus*: a regulatory link between metabolism and virulence gene expression.. J Bacteriol.

[67] Loh E, Dussurget O, Gripenland J, Vaitkevicius K, Tiensuu T (2009). A trans-acting riboswitch controls expression of the virulence regulator PrfA in *Listeria monocytogenes*.. Cell.

[68] Mulhbacher J, Brouillette E, Allard M, Fortier LC, Malouin F (2010). Novel Riboswitch Ligand Analogs as Selective Inhibitors of Guanine-Related Metabolic Pathways.. PLoS Pathog.

[69] Wurtzel O, Sapra R, Chen F, Zhu Y, Simmons BA (2009). A single-base resolution map of an archaeal transcriptome.. Genome Res.

[70] Brantl S (2007). Regulatory mechanisms employed by cis-encoded antisense RNAs.. Curr Opin Microbiol.

[71] Georg J, Voss B, Scholz I, Mitschke J, Wilde A (2009). Evidence for a major role of antisense RNAs in cyanobacterial gene regulation.. Mol Syst Biol.

[72] Yelin R, Dahary D, Sorek R, Levanon EY, Goldstein O (2003). Widespread occurrence of antisense transcription in the human genome.. Nat Biotechnol.

[73] Katayama S, Tomaru Y, Kasukawa T, Waki K, Nakanishi M (2005). Antisense transcription in the mammalian transcriptome.. Science.

[74] Voyich JM, Braughton KR, Sturdevant DE, Whitney AR, Said-Salim B (2005). Insights into mechanisms used by *Staphylococcus aureus* to avoid destruction by human neutrophils.. J Immunol.

[75] Brantl S (2002). Antisense-RNA regulation and RNA interference.. Biochim Biophys Acta.

[76] Nagy Z, Chandler M (2004). Regulation of transposition in bacteria.. Res Microbiol.

[77] Takeuchi F, Watanabe S, Baba T, Yuzawa H, Ito T (2005). Whole-genome sequencing of *Staphylococcus haemolyticus* uncovers the extreme plasticity of its genome and the evolution of human-colonizing staphylococcal species.. J Bacteriol.

[78] Simons RW, Kleckner N (1983). Translational control of IS10 transposition.. Cell.

[79] Wagner EG, Altuvia S, Romby P (2002). Antisense RNAs in bacteria and their genetic elements.. Adv Genet.

[80] Chesneau O, Lailler R, Derbise A, El Solh N (1999). Transposition of IS1181 in the genomes of *Staphylococcus* and *Listeria*.. FEMS Microbiol Lett.

[81] Mandin P, Repoila F, Vergassola M, Geissmann T, Cossart P (2007). Identification of new noncoding RNAs in *Listeria monocytogenes* and prediction of mRNA targets.. Nucleic Acids Res.

[82] Sridhar J, Rafi ZA (2007). Identification of novel genomic islands associated with small RNAs.. In Silico Biol.

[83] Balbontin R, Figueroa-Bossi N, Casadesus J, Bossi L (2008). Insertion hot spot for horizontally acquired DNA within a bidirectional small-RNA locus in *Salmonella enterica*.. J Bacteriol.

[84] Chevalier C, Boisset S, Romilly C, Masquida B, Fechter P (2010). *Staphylococcus aureus* RNAIII binds to two distant regions of *coa* mRNA to arrest translation and promote mRNA degradation.. PloSPathogen.

[85] Rosenblad MA, Larsen N, Samuelsson T, Zwieb C (2009). Kinship in the SRP RNA family.. RNA Biol.

[86] Li H, Ruan J, Durbin R (2008). Mapping short DNA sequencing reads and calling variants using mapping quality scores.. Genome Res.

[87] Rutherford K, Parkhill J, Crook J, Horsnell T, Rice P (2000). Artemis: sequence visualization and annotation.. Bioinformatics.

[88] Chien Y, Manna AC, Projan SJ, Cheung AL (1999). SarA, a global regulator of virulence determinants in *Staphylococcus aureus*, binds to a conserved motif essential for *sar*-dependent gene regulation.. J Biol Chem.

[89] Rivas E, Eddy SR (2001). Noncoding RNA gene detection using comparative sequence analysis.. BMC Bioinformatics.

[90] Cros MJ, Sallet E, Moisan A, Cierco-Ayrolles C, Gaspin C (2007). Visualizing and exploring genomic information for non-protein-coding RNA identification using ApolloRNA.. Nature protocols.

[91] Baba T, Kuwahara-Arai K, Uchiyama I, Takeuchi F, Ito T (2009). Complete genome sequence of *Macrococcus caseolyticus* strain JCSCS5402, [corrected] reflecting the ancestral genome of the human-pathogenic staphylococci.. J Bacteriol.

