# Supplementary figures and images for: Cartography of Methicillin-Resistant S. aureus Transcripts: Detection, Orientation and Temporal Expression during Growth Phase and Stress Conditions
Source: PLoS One. 2010 May 20;5(5):e10725. doi: 10.1371/journal.pone.0010725 (PMC2873960; doi:10.1371/journal.pone.0010725)

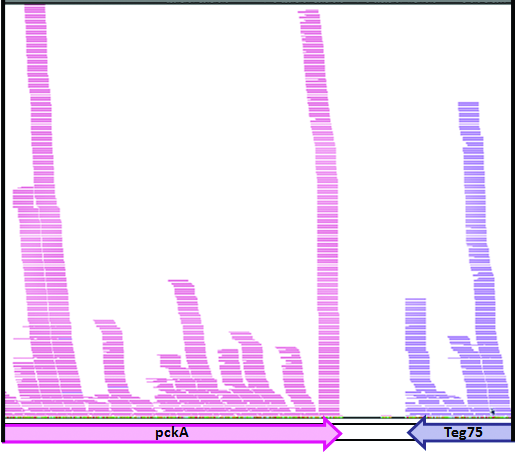

Supplement: Figure S1 — Reads mapping onto the S. aureus genomic sequence from dir-mRNA-SEQ protocol. (1.51 MB TIF) [file pone.0010725.s001.tif]
