# Supplementary material for: Cartography of Methicillin-Resistant S. aureus Transcripts: Detection, Orientation and Temporal Expression during Growth Phase and Stress Conditions
Source: PLoS One. 2010 May 20;5(5):e10725. doi: 10.1371/journal.pone.0010725 (PMC2873960; doi:10.1371/journal.pone.0010725)
Supplement: Figure S2 — Secondary structures of some sRNAs bona fide based on in silico analysis. (0.14 MB DOC) [file pone.0010725.s002.doc]

**Supporting Figure S2**
