# Supplementary material for: Cartography of Methicillin-Resistant S. aureus Transcripts: Detection, Orientation and Temporal Expression during Growth Phase and Stress Conditions
Source: PLoS One. 2010 May 20;5(5):e10725. doi: 10.1371/journal.pone.0010725 (PMC2873960; doi:10.1371/journal.pone.0010725)
Supplement: Table S3 — Characteristics of oligonucleotides used in the RT-qPCR assay. (0.06 MB DOC) [file pone.0010725.s005.doc]

**Supporting Table S3**

| **Primers** | | **Sequence (5’ -> 3’)** | **Length (nt)** | **Final concentration (µM)** |
| --- | --- | --- | --- | --- |
| Teg1 | Forward | GTTGGTTTTAGCACCGTGCTATAAA | 25 | 0.2 |
|  | Reverse | TCATCACTGAATCTCAACTAACACATAACA | 30 |  |
| Teg4 | Forward | TTAACCGAAAGCCTGAATGC | 20 | 0.2 |
|  | Reverse | GCATATGGATTTCACTGGTGTT | 22 |  |
| Teg17 | Forward | TGGTCACTTTAAATTATAGAGG | 22 | 0.5 |
|  | Reverse | GGGAAGGTAAAACTTCCTGCT | 21 |  |
| Teg18 | Forward | TCACCTCGTTGATTATGTTC | 20 | 0.2 |
|  | Reverse | TAACGGTCAGCTTTATTGAA | 20 |  |
| Teg19b | Forward | TAACTCTTGAAACACAAAAAGGGCAA | 26 | 0.2 |
|  | Reverse | ACTCCTAGAAAGGACTAGCATGTCTGA | 27 |  |
| Teg21 | Forward | CCTCCTGGTTGATTATTCTCG | 21 | 0.2 |
|  | Reverse | CGCAGATGCTACTACTTATCTTAACA | 26 |  |
| Teg24 | Forward | AGATGTTGTGATGTAACTTGAACGA | 25 | 0.2 |
|  | Reverse | CGATTAAACAAAGGGGTAGAGC | 22 |  |
| Teg26 | Forward | ATCTCCTCCTATAATTCGATATTCA | 25 | 0.2 |
|  | Reverse | ACGTCTTTGGTAACAAGCCA | 20 |  |
| Teg28 | Forward | TTTTCATCTGTAATGTGAATACTTA | 25 | 0.2 |
|  | Reverse | ATGTGCAAACAAAGTAATCG | 20 |  |
| Teg35 | Forward | GTGTTCGATATCAGAGAGCTTGTGG | 25 | 0.2 |
|  | Reverse | TCCCTACAAGGGACGTGGTCTAC | 23 |  |
| Teg38 | Forward | AACACGTTGGTGTATGTAATTAATGT | 26 | 0.2 |
|  | Reverse | CAATAAGATGTGATGGTAAGCTAGA | 25 |  |
| Teg42 | Forward | GTAGCGGTTCCCTGTACTCGAAA | 23 | 0.5 |
|  | Reverse | GGACCGAAATCTTCAAACACTACGT | 25 |  |
| Teg45 | Forward | ATTAAATTTATACGATGCAGAGAGTGTACG | 30 | 0.2 |
|  | Reverse | CCACTCTGAATTTAGCGAATAACATTAATA | 30 |  |
| Teg47 | Forward | AGCTTTGAATACTTAAAATTCTCTTGC | 27 | 0.2 |
|  | Reverse | TTGCGTTGGACAAAATACTC | 20 |  |
| Teg55 | Forward | TGTTTCACAACAAAAACGTGA | 21 | 0.5 |
|  | Reverse | ATAATTTCAAAAATGACTATGCAACA | 26 |  |
| Teg56 | Forward | ACATTTCTACGTGAATGCATACACAAT | 27 | 0.2 |
|  | Reverse | GTTTAATATTGATAACGGATTGTCTTTCC | 29 |  |
| Teg57 | Forward | TTTTTGAATGTACGTGGAAGCGT | 23 | 0.2 |
|  | Reverse | TAACCCCCTAAAGTGATATCGTTTCTA | 27 |  |
| Teg60 | Forward | TTGGCGTTTGCTGCTTATATATTATTT | 27 | 0.5 |
|  | Reverse | TGCCTCTTTGATTTAATATGATCTTATCA | 29 |  |
| Teg61 | Forward | CGTGATTGTGTAAAGGGTGC | 20 | 0.2 |
|  | Reverse | TGGTTAATTACCTCCATAGCAAAC | 24 |  |
| Teg69 | Forward | TCCGTGTTGCCACCCTCATT | 20 | 0.2 |
|  | Reverse | CGTACGTTAAACGTTTTGAGTGGG | 24 |  |
| Teg70 | Forward | CGTGGTACCACCTATATTCAAGAAGG | 26 | 0.2 |
|  | Reverse | TGATTTTTACTGAAGGTAGCCCACA | 25 |  |
| Teg72 | Forward | CAACAATTTATCTAAATCGCCCCTC | 25 | 0.2 |
|  | Forward | TGCGCTTAAAATAGATGCTTAATTAAAA | 28 |  |
| Teg73 | Reverse | TCACAAAAATTGTGCACTCA | 20 | 0.2 |
|  | Forward | TGCTGAGAGCTAGTGTTAAGGA | 22 |  |
| Teg76 | Reverse | TAAAGAATATGGCTTTAGAAGTTTCTACCA | 30 | 0.2 |
|  | Forward | ACGAATTTGTCATGCAATCAATGTA | 25 |  |
| Teg91 | Forward | GGCTTTTATGGGAAATGAATATTATTG | 27 | 0.5 |
|  | Reverse | TTTATAATAACAAAACAAATTTTGCTCGA | 29 |  |
| Teg2pl | Forward | CGAAAATAATAAAGGGAAAAT | 21 | 0.2 |
|  | Reverse | TCGTTGACATGTATAATTTTGA | 22 |  |
